# Cardioembolic stroke versus embolic stroke of undetermined source: early severity and long-term outcomes in a prospective cohort

**DOI:** 10.1186/s12883-025-04494-w

**Published:** 2025-12-01

**Authors:** Priyanka Boettger, Jamschid Sedighi, Martin Juenemann, Michael Buerke, Omar Alhaj Omar

**Affiliations:** 1 Department of Cardiology, Angiology and Critical Care Medicine, Justus Liebig University, Klinikstrasse 33, Giessen, Hessen 35392 Germany; 2 Department of Neurology, Justus Liebig University, Klinikstrasse 33, Giessen, Hessen 35392 Germany; 3 Department of Cardiology, Angiology and Critical Care Medicine, St. Marien Hospital, Kampenstrasse 51, Siegen, Nord Rhine-Westphalia 57072 Germany

**Keywords:** Embolic stroke of undetermined source, Atrial fibrillation, Cardioembolic stroke, Stroke severity, Sex differences, Atrial cardiopathy, Functional outcome

## Abstract

**Background:**

Embolic stroke of undetermined source (ESUS) constitutes a substantial proportion of ischemic strokes. Its distinction from cardioembolic stroke (CES) may carry prognostic implications. This study aimed to compare early and long-term outcomes between ESUS and CES.

**Methods:**

We conducted a prospective cohort study of patients with ESUS and CES who underwent standardized diagnostic evaluation and prolonged cardiac monitoring. Stroke severity was assessed with the NIHSS, and functional outcome with the mRS at discharge, 30 days, and 12 months. ESUS patients were additionally evaluated for short-duration atrial fibrillation.

**Results:**

During a 6-month prospective enrollment period, 771 patients with suspected stroke were screened, of whom 529 had ischemic stroke confirmed by neuroimaging. After applying ESUS criteria, 98 patients were classified as ESUS and 209 as CES (total study cohort, *n* = 307; 41% women; mean age 72 ± 9 years). Patients with ESUS presented with significantly lower stroke severity and achieved better functional outcomes than those with CES. The mean NIHSS score on admission was 6 (95% CI, 5–7) in the ESUS group and 11 (95% CI, 10–12) in the cardioembolic group ( *p*< 0.001). At discharge, the mean mRS score was 2.1 (95% CI, 1.8–2.4) for ESUS and 3.8 (95% CI, 3.5–4.1) for CES (*p* < 0.001). A favorable outcome (mRS 0–2) was more common in ESUS (68% vs. 34%; *p* < 0.001). In-hospital mortality occurred in 10.0% of cardioembolic patients and in none of the ESUS patients (*p* = 0.02). At 12 months, ESUS patients continued to show more favorable recovery, with higher rates of functional independence (60% vs. 28%; *p* < 0.001) and lower cumulative mortality (7.1% vs. 21.5%; *p*< 0.001).

**Conclusions:**

Compared with CES, ESUS was associated with less severe neurological deficits and more favorable outcomes, both early and at 12 months, including lower mortality and better functional recovery.

**Supplementary Information:**

The online version contains supplementary material available at 10.1186/s12883-025-04494-w.

## Introduction

Embolic stroke of undetermined source (ESUS) [1] was introduced to refine the classification of cryptogenic strokes with presumed embolic origin by standardizing the exclusion of major cardioembolic sources, significant large-artery stenosis, and small-vessel disease. Although the ESUS construct was conceptualized to unify a heterogeneous group for therapeutic studies, subsequent trials such as NAVIGATE ESUS [2] and RE-SPECT ESUS [3] did not demonstrate superiority of anticoagulation over antiplatelet therapy, challenging the initial therapeutic premise. Nevertheless, observational studies have highlighted that ESUS patients often exhibit features suggestive of covert embolic mechanisms, including atrial cardiopathy and subclinical atrial fibrillation [4]. Comparative analyses of ESUS and CES regarding clinical severity and functional outcomes remain limited. Given the potential overlap in embolic substrates, a better understanding of stroke characteristics across these entities may aid in refining diagnostic strategies and informing secondary prevention. In this prospective study, we aimed to characterize stroke severity and early functional outcomes in patients with ESUS compared to those with CES, with a focus on the impact of short-duration atrial fibrillation detected during hospitalization and age- and sex-related differences in functional outcomes.

## Methods

### Study design and population

**Fig. 1:**
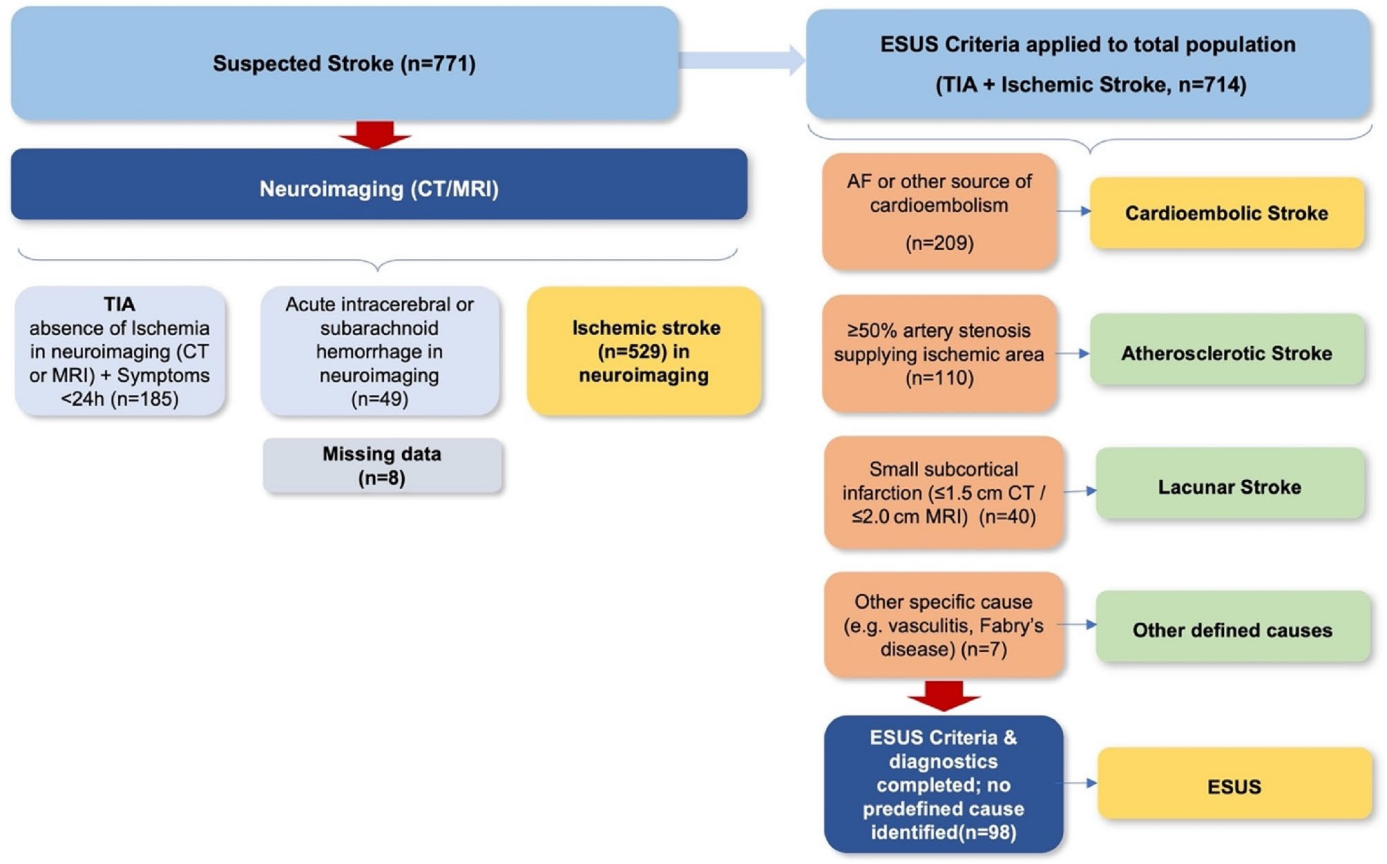
Study population and classification of stroke subtypes. From a total of 714 patients, 185 were diagnosed with transient ischemic attack (TIA). Among the remaining 529 patients with ischemic stroke, 209 were classified as cardioembolic stroke (CES), 110 as atherosclerotic stroke, 163 as cryptogenic (among them 98 fulfilled ESUS diagnostics and criteria) and 40 as lacunar stroke, and 7 as other defined causes. A total of 163 patients were categorized as cryptogenic stroke, of whom 98 fulfilled criteria for embolic stroke of undetermined source (ESUS)

This prospective cohort study was conducted at a large academic hospital over a six-month period. All consecutive patients aged ≥ 18 years with acute ischemic stroke or transient ischemic attack (TIA) confirmed by neuroimaging were eligible. Patients with hemorrhagic stroke or hospital stays shorter than 24 h were excluded. Written informed consent was obtained from all participants or their legal representatives. The study protocol was approved by the local ethics committee (Westfalen-Lippe Medical Association). The inclusion and exclusion process, as well as the final cohort composition, are summarized in the study flow diagram (Figure 1).

### Stroke classification

Stroke etiology was classified according to the Trial of Org 10,172 in Acute Stroke Treatment (TOAST) criteria [5] and the original definition of embolic stroke of undetermined source (ESUS) by Hart et al. [1].

 Transient ischemic attack (TIA) was defined according to the tissue-based American Heart Association/American Stroke Association (AHA/ASA) definition [6, 7] as a transient episode of neurological dysfunction without evidence of acute infarction on brain imaging [8]. Patients presenting with TIA but without an acute ischemic lesion on CT or MRI were therefore not classified as ESUS and were excluded from the present analysis.

ESUS [1] was defined as an imaging-confirmed, (1) non-lacunar infarction (cortical, cortico-subcortical, cerebellar, or brainstem; > 1.5 cm on CT or > 2 cm on DWI) in the absence of (2) major-risk cardioembolic sources, (3) extracranial or intracranial atherosclerosis causing ≥ 50% luminal stenosis according to NASCET criteria in the artery supplying the infarcted territory [9], or (3) other determined stroke etiologies [10].

 Cardioembolic stroke (CES) [11] was defined by the presence of high-risk cardiac sources, including atrial fibrillation (AF) or atrial flutter (documented on ECG, Holter monitoring, or continuous telemetry), intracardiac thrombus, mechanical prosthetic valves, infective endocarditis, recent myocardial infarction (< 4 weeks), or left ventricular ejection fraction < 35%. In line with AHA/ACC/HRS guidelines [12, 13], HRS/EHRA/ECAS and EHRA/HRS/APHRS/LAHRS consensus statements [14–16], AF was only classified as such if the arrhythmia lasted ≥ 30 s [8]. Consequently, patients with shorter atrial arrhythmia episodes were not reclassified as CES but were analyzed as part of the ESUS group, representing a potential intermediate phenotype within the ESUS spectrum, consistent with our previous methodology [17].

### Diagnostic workup and clinical assessment

All patients underwent standardized diagnostic evaluation including brain imaging computed tomography (CT) and/or magnetic resonance imaging (MRI), extracranial and intracranial vascular imaging (ultrasound and/or CT/MR angiography), transthoracic echocardiography (TTE), transesophageal echocardiography (TEE) where indicated, and continuous cardiac rhythm monitoring with stroke-unit telemetry and at least 24-hour Holter ECG. Neuroimaging was performed in all patients to confirm the diagnosis: MRI in 664 patients (93.0%), CT in 264 patients (37.0%), and both MRI and CT in 104 patients (14.5%). Vascular imaging was completed in 678 (95%) of all patients, predominantly by carotid and vertebral duplex ultrasound in 645 (95.1%) and MR angiography in 616 (91.2%). Transthoracic echocardiography (TTE) was performed in 343 (48%) and transesophageal echocardiography (TEE) in 186 (26%) patients, with a higher TEE rate in the ESUS subgroup (38.8% vs. 17.4%, *p* < 0.001). Continuous cardiac monitoring was performed in all patients for a minimum of 24 h during the acute stroke phase (median telemetry duration 46 h; IQR 30–72 h). Stroke-unit telemetry was completed in 650 patients (91%; 96% ESUS [n = 94], 90% CES [n = 188]), 24-hour Holter ECG in 321 (45%; 60% ESUS [n = 59] vs. 43% CES [n = 90]) and extended external (72 h–7 days) or implantable loop monitoring in selected cases. Rhythm events were automatically detected and independently adjudicated by a stroke fellow and a cardiologist, with consensus resolution in case of discrepancy.

Follow-up assessments were performed at discharge, 30 days, and 12 months after the index event. Data collection included in-person visits at our outpatient clinic whenever feasible; when this was not possible, questionnaires were mailed and structured telephone interviews conducted. Pre-specified subgroup analyses were performed by sex and age (< 65 vs. ≥ 65 years; < 80 vs. ≥ 80 years).

### Clinical assessments

Neurological severity and functional outcome were assessed using validated instruments. Assessments were performed at admission, discharge, 30 days, and 12 months, in accordance with the study protocol. Stroke severity was measured with the National Institutes of Health Stroke Scale (NIHSS) [18], and functional outcome with the mRS [19]. Additionally, at 12 months, health-related quality of life was evaluated using the EQ-5D-L questionnaire [20], and independence in daily living with the Barthel Index [21] were assessed in person. If in-person visits were not feasible, the EQ-5D-5L questionnaire [20, 22–24] was mailed to patients and functional outcome was assessed by structured telephone interviews using the validated telephone version of the mRS [25–30]. None of these instruments were developed for this study; all have been previously validated and widely applied in stroke research.

### Statistical analysis

Continuous variables are reported as means ± standard deviation (SD) or medians with interquartile ranges (IQR), as appropriate. Categorical variables are presented as counts and percentages. Group comparisons were performed using Student’s t test or Mann–Whitney U test for continuous variables and chi-square test or Fisher’s exact test for categorical variables, as appropriate. Functional outcomes measured by mRS and stroke severity measured by NIHSS were compared between ESUS and CES at discharge, 30 days, and 12 months. Mortality rates were analyzed using chi-square tests. Pre-specified subgroup analyses were performed according to sex and age (< 65 vs. ≥ 65 years; < 80 vs. ≥ 80 years), and interactions between stroke subtype and these variables were formally tested. To assess the robustness of outcome differences, a sensitivity analysis stratified by cardiac monitoring intensity (telemetry only vs. ≥ 24-hour Holter vs. extended/implantable monitoring) was performed. NIHSS was included only in sensitivity analyses, as adjustment in the main model could constitute overadjustment given its mediator role between subtype and outcome. A sensitivity model including baseline NIHSS and a subgroup analysis stratified by stroke severity (NIHSS ≤ 5 vs. > 5) were performed to test the stability of associations.

Multivariable linear regression (for mRS and NIHSS) and logistic regression (for mortality and functional independence defined as mRS 0–2) were performed to adjust for potential confounders, including age, sex, vascular risk factors, and atrial fibrillation status. Results are reported as adjusted β coefficients or odds ratios (OR) with 95% confidence intervals (CI). All tests were two-sided, and p-values < 0.05 were considered statistically significant. Statistical analyses were conducted using SPSS Statistics, version 28 (IBM Corp.).

## Results

**Fig. 2:**
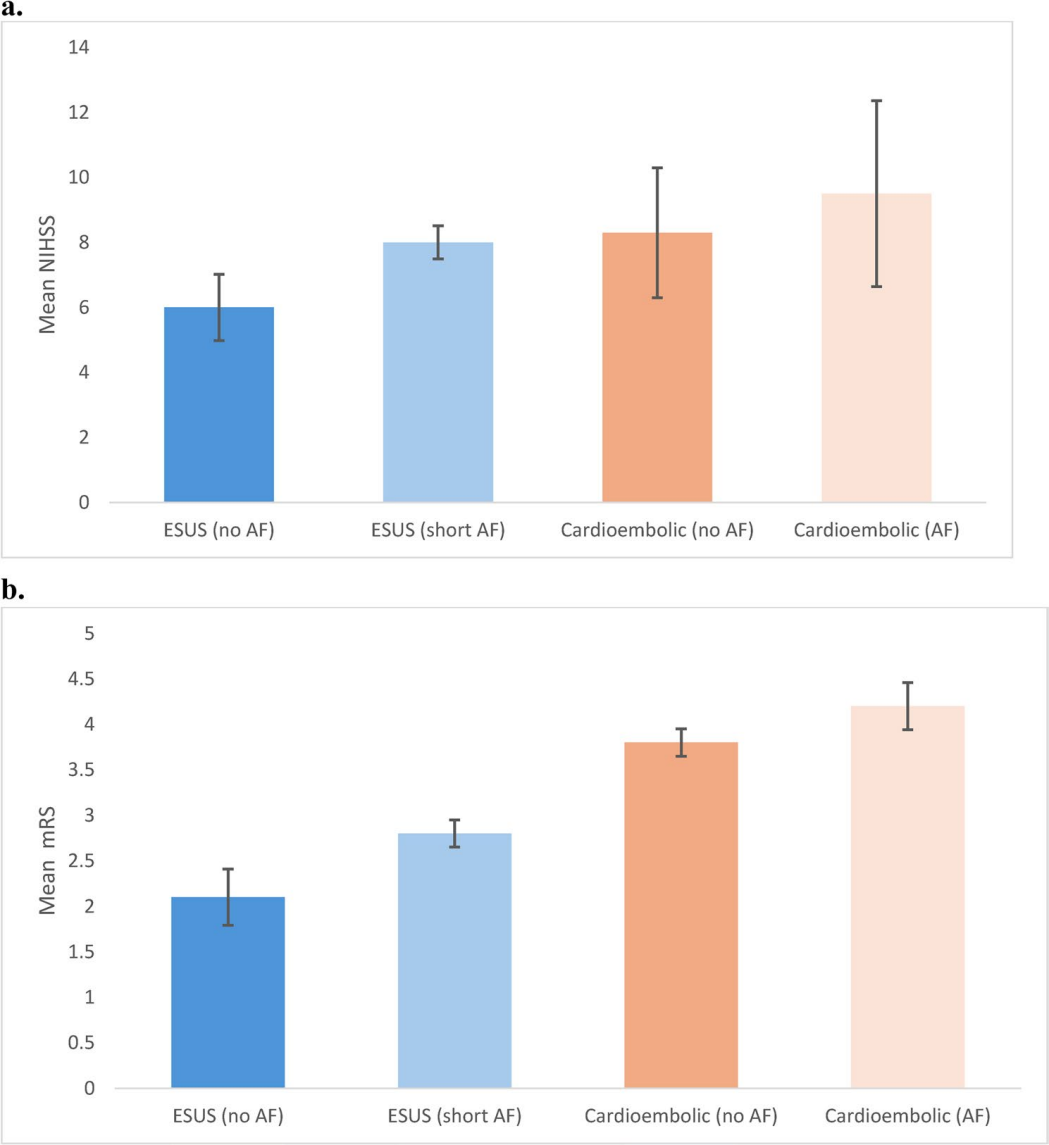
Stroke severity by subtype and atrial fibrillation status.** A** Bar plots display mean stroke severity at admission, measured by the National Institutes of Health Stroke Scale (NIHSS), across four patient groups: ESUS without atrial fibrillation (AF), ESUS with short-duration AF, cardioembolic stroke without documented AF, and cardioembolic stroke with AF. Error bars represent 95% confidence intervals. Stroke severity was lowest in ESUS without AF and highest in cardioembolic stroke with AF, with intermediate values in ESUS with short-duration AF. **B** Bar plots show mean modified Rankin Scale (mRS) scores at discharge, indicating functional outcome, for the same four patient groups. Error bars indicate 95% confidence intervals. Functional outcomes followed a similar trend to NIHSS, with best outcomes in ESUS without AF and worst in cardioembolic stroke with AF. Patients with short-duration AF in the ESUS group had intermediate mRS scores, supporting a potential link to cardioembolic stroke mechanisms

A total of 714 patients with acute ischemic stroke or TIA were enrolled during the study period. 185 TIA patients were excluded from the study. Of the remaining population, 98 patients (14.9%) fulfilled criteria for ESUS, and 209 patients (31.8%) were classified as CES (Fig. 2a). Patients with ESUS were younger than those with CES. The mean age was 67 years (95% CI, 65 to 69) for ESUS and 75 years (95% CI, 73 to 77) for CES (*p* < 0.001). Female patients were less frequent among ESUS cases (38.8%) compared to CES (43.1%), although the difference was not statistically significant (*p* = 0.46). In both stroke subtypes, women were older than men. In the ESUS cohort, the mean age was 71 years for women versus 65 years for men (*p *= 0.02). In the cardioembolic cohort, women had a mean age of 77 years compared to 72 years in men (*p* < 0.001). Hypertension was common across both groups (72.2% vs. 74.6%; *p* = 0.64), as was obesity (54.2% vs. 45.3%; *p* = 0.11). Further baseline characteristics can be found in Table 1. Premorbid CHA₂DS₂-VASc scores were comparable between ESUS and cardioembolic patients (mean 4.2 ± 1.1 vs. 4.6 ± 1.3; *p* = 0.08). To further investigate possible sources of embolism beyond baseline demographics, an exploratory analysis of cardiac and vascular findings was performed during in-hospital evaluation (Table 2).

**Table 1:** Baseline characteristics of patients with ischemic stroke, stratified by stroke subtype

**Variable**	**Total stroke**	**ESUS**	**CES**
n	n=714	n=98	n=209
Age, mean ± SD	71 ± 9.2	67 ± 9.8	75 ± 7.9
Age, median (IQR)	74 (67–81)	70 (64–77)	77 (71–83)
NIHSS at admission, median (IQR)	7 (3–11)	6 (3–9)	11 (6–16)
NIHSS at discharge, median (IQR)	3 (1–5)	2 (1–3)	5 (3–7)
Previous TIA/stroke	181 (25.4%)	11 (11.2%)	55 (26.3%)
BMI, mean ± SD	29.1 ± 4.2	30.4 ± 4.1	29.5 ± 4.4
Obesity (BMI>30 kg/m2)	334 (46.8%)	53 (54.2%)	106 (50.7%)
Hypertension	537 (75.2%)	71 (72.2%)	156 (74.6%)
Diabetes mellitus	212	31 (31.6%)	66 (31.6%)
Atrial fibrillation	163 (22.8%)	0 (0.0%)	101 (48.3%)
CAD	191 (26.8%)	27 (27.5%)	64 (30.6%)
Artificial heart valve	41 (5.7%)	0 (0%)	30 (14.3%)
Heart failure	66 (9%)	6 (6.1%)	40 (19.3%)
Hypercholesterinemia	274 (38.4%)	42 (42.9%)	66 (31.6%)
SUM, mean ± SD (h)	37.1 ± 6.8	36.0 ± 6.2	42.5 ± 6.9
SUM	651 (91.2%)	94 (96.0%)	191 (91.4%)

Values are presented as mean ± SD or median (interquartile range) for continuous variables, and as n (%) for categorical variables.* NIHSS* National Institutes of Health Stroke Scale, *TIA* transient ischemic attack, *BMI* Body mass index, *SD* Standard deviation, *IQR* Interquartile range, *h* hours, *ESUS* Embolic stroke of undetermined source, *CAD* Coronary artery disease, *SUM* Stroke unit monitoring

**Table 2:** Potential embolic sources identified during in-hospital evaluation in ESUS

**Category**	**Embolic source/parameter**	**Primary detection modality**	**ESUS, n (%)**	**CES, n (%)**	***P value***
***Aortic and large-artery sources***	Complex aortic arch atherosclerosis	TEE (38 [38.8 %]) ± CTA/MRA (performed in 93 [94.9 %])	3 (3.1)	4 (1.9)	0.42
	Non-complex aortic arch atherosclerosis	CTA/MRA ± TEE	8 (8.2)	18 (8.6)	0.91
	Aortic arch anatomical variants (elongation, kinking, aneurysmal dilation)	CTA/MRA	3 (3.1)	7 (3.3)	0.94
	Non-occlusive carotid/vertebral atherosclerotic plaque (< 50 % stenosis)	CTA/MRA or duplex ultrasound	37 (37.8)	69 (33.0)	0.38
***Cardiac sources***	Short-duration atrial fibrillation (< 30 s)	Stroke-unit telemetry (94 [96.0 %]) ± Holter ≥ 24 h (59 [60.2 %])	35 (35.7)	88 (42.1) †	0.23
	– 15–29 s episodes		18 (18.4)	42 (20.1)	
	– 0–14 s episodes		17 (17.3)	46 (22.0)	
	Clinical AF (≥ 30 s or previously diagnosed)	ECG/telemetry/history	0 (0)	94 (45.0)	**< 0.001**
	Atrial cardiopathy	TTE (51 [52.0 %])	20 (20.4)	37 (42.1)	**0.002**
	LVEF >35 % with regional hypokinesia)	TTE (51 [52.0 %])	5 (5.1)	16 (18.2)	**0.005**
	LVEF < 35 %	TTE	0 (0)	40 (19.3)	**< 0.001**
***Right-to-left shunt***	Patent foramen ovale/interatrial shunt	TEE (38 [38.8 %])	11 (11.2)	4 (4.5)	**0.04**
***Systemic/hematologic***	Active malignancy or pro-thrombotic state	Clinical/laboratory assessment/patient history	3 (3.1)	6 (2.9)	0.91

Values are presented in n (%). Neuroimaging was performed in all patients. Vascular imaging extending to the aortic arch was available in 93/98 (94.9%) using CTA or MRA (± duplex). TTE was performed in 51/98 (52.0%), and TEE in 38/98 (38.8%). Continuous rhythm monitoring on the stroke unit was available in 94/98 (96.0%), and Holter ECG ≥ 24 h in 59/98 (60.2%)

*Complex aortic arch atherosclerosis* = plaque ≥ 4 mm, ulcerated, or mobile; *Non-Complex aortic arch atherosclerosis* = plaque <4 mm; *Non-occlusive carotid/vertebral plaque* = luminal stenosis < 50 % ipsilateral to the infarct; *Atrial cardiopathy* = left atrial diameter ≥ 42 mm or LAVI >34 mL/m²; *Short-duration atrial fibrillation* = AF episodes < 30 s on telemetry or Holter.; *LVEF >35 % with regional hypokinesia* was considered an indirect indicator of previous ischemic injury, significant coronary artery disease, or myocarditis; no systematic cardiac MRI or coronary angiography was performed to further clarify etiology. Exclusions: Patients with >50% arterial stenosis with an infarction in the supplying area, left-ventricular thrombus, infective endocarditis, LVEF<35%, AF above 30 seconds or vasculitis were excluded according to ESUS criteria [1]

*AF* Atrial fibrillation, *AAA* Aortic arch atherosclerosis, *CES* Cardioembolic stroke, *CI* Confidence interval, *CTA* Computed tomography angiography, *ESUS* Embolic stroke of undetermined source, *LA* Left atrium, *LAVI* Left atrial volume index, *LVEF* Left ventricular ejection fraction, *MRI* Magnetic resonance imaging, *MRA* Magnetic resonance angiography, *NIHSS* National Institutes of Health Stroke Scale, *OR* Odds ratio, *PFO* Patent foramen ovale, *SD* Standard deviation, *TEE* Transesophageal echocardiography, *TTE* Transthoracic echocardiography

### Exploratory analysis of potential embolic substrates in ESUS and CES 

To further elucidate the underlying mechanisms of embolism, we performed an exploratory comparison of potential cardiac and vascular embolic substrates detected during standardized in-hospital evaluation (Table 2). TTE and TEE were available in approximately half of all patients, with no significant difference in testing frequency between groups. Vascular imaging extending from the intracranial circulation to the aortic arch was obtained in nearly all patients (94.9%).

Among individuals with ESUS, non-occlusive carotid or vertebral plaques (< 50% stenosis) were the most frequent potential source (37.8%) compared with 33.0% in CES (*p* = 0.46). High-risk plaque morphology—defined by irregular surface or ulceration—was observed in 8.2% and 10.1%, respectively (*p* = 0.62). Although these differences were not statistically significant, substenotic atherosclerosis was common in both ESUS and CES. Non-complex aortic arch atherosclerosis was present in 8.2% and complex plaques (≥ 4 mm thickness, ulceration, or mobile components) in 3.1% of ESUS cases. Atrial cardiopathy, characterized by left atrial enlargement or increased left atrial volume index, was observed in 20.4% of ESUS compared with 42.1% of CES patients (*p* = 0.002). Short-duration atrial fibrillation (< 30 s) occurred in 35.7% of ESUS and 79.3% of CES (*p* < 0.001). Patent foramen ovale (PFO) was more prevalent in ESUS (11.2%) than CES (4.5%; *p* = 0.04), whereas regional left-ventricular hypokinesia with preserved systolic function (LVEF > 35%) was identified in 5.1% versus 18.2%, respectively (*p* = 0.005), consistent with prior ischemic injury, myocarditis, or unrecognized coronary artery disease.

### Premorbid vascular and cardiac therapy

Premorbid treatment profiles differed substantially between ESUS and CES (Table 3). A history of hypertension was more common in CES than in ESUS (51.7% vs. 29.6%; *p* = 0.001), although the relative use of antihypertensive subclasses—including ACE inhibitors, ARBs, beta blockers, and calcium channel blockers—was comparable between groups. Heart failure was reported in 19.3% of CES but only 6.1% of ESUS patients (*p* = 0.003). Oral anticoagulant therapy prior to stroke onset occurred exclusively in CES (18.8%; *p* < 0.001), consistent with the higher prevalence of atrial fibrillation or mechanical heart valves. A history of vascular disease was present in approximately half of ESUS patients (49.0%) and two-thirds of CES patients (67.0%; *p* = 0.004). Premorbid antiplatelet use differed markedly: aspirin was used by nearly all ESUS patients (97.9%) but by only half of those with CES (48.3%; *p* < 0.001), while P2Y₁₂ inhibitor use remained low and similar across groups (6.3% vs. 9.1%; *p* = 0.46).

**Table 3:** Premorbid vascular and cardiac therapy in ESUS and CES

***Condition/Drug class***	***ESUS (n = 98)***	***CES (n = 209)***	***p value***
***Hypertension***	29 (29.6)	108 (51.7)	0.001
*ACE inhibitors*	11 (37.9)	42 (38.9)	0.92
*ARBs*	10 (34.5)	39 (36.1)	0.86
*Beta blockers*	13 (44.8)	49 (45.4)	0.94
*Calcium channel blockers*	9 (31.0)	32 (29.6)	0.88
*Thiazide diuretics*	7 (24.1)	26 (24.1)	0.99
*Loop diuretics*	3 (10.3)	12 (11.1)	0.89
*Mineralocorticoid antagonists*	2 (6.9)	8 (7.4)	0.91
***Heart failure***	6 (6.1)	40 (19.3)	**0.003**
***Atrial fibrillation/mechanical valve***	0 (0.0)	83 (18.8)	**<0.001**
*NOAC*	0 (0.0)	60 (72.3)	**<0.001**
*Vitamin K antagonist*	0 (0.0)	12 (20.0)	**<0.001**
***Premorbid vascular disease***	48 (49.0)	140 (67.0)	**0.004**
*Aspirin*	47 (97.9)	101 (48.3)	**<0.001**
*P2Y₁₂ inhibitor (clopidogrel, ticagrelor, or prasugrel)*	3 (6.3)	19 (9.1)	0.46

Percentages refer to the proportion of patients within each relevant subgroup who received the respective medications prior to stroke onset. Values are presented in n (%)*, ACE* Angiotensin-converting enzyme, *ARB* Angiotensin receptor blocker, *CES* Cardioembolic stroke, *ESUS* Embolic stroke of undetermined source, *NOAC* Non-vitamin K oral anticoagulant, *P2Y*₁₂ Purinergic receptor subtype Y12 inhibitor

### Antithrombotic regimens at discharge

Discharge antithrombotic therapy differed markedly between ESUS and CES (Table 4). Among ESUS patients, nearly all were discharged on single antiplatelet therapy (90.8%), and three patients (3.1%) received a direct oral anticoagulant (DOAC) after detection of recurrent short-duration atrial fibrillation during continuous monitoring. None received vitamin K antagonists. In contrast, oral anticoagulation was prescribed in 78.0% of CES patients—predominantly DOACs (66.5%)—whereas dual antiplatelet therapy (DAPT) was used in 10.0% and single antiplatelet therapy in 8.6%. Between-group differences were significant for oral anticoagulation (78.0% vs. 3.1%; *p* < 0.001) and single antiplatelet therapy (90.8% vs. 8.6%; *p* < 0.001), while DAPT use showed no significant difference (10.0% vs. 6.1%; *p* = 0.22). No ESUS patient was discharged without antithrombotic medication, compared with 3.3% of CES patients (*p* = 0.18).

**Table 4:** Antithrombotic therapy at discharge

**Regimen**	**ESUS (n = 98)**	**CES (n = 209)**	***p value***
***Single antiplatelet (ASA or P2Y₁₂ inhibitor)***	89 (90.8)	18 (8.6)	< 0.001
***Dual antiplatelet therapy (ASA + P2Y₁₂ inhibitor)***	6 (6.1)	21 (10.0)	0.22
***Oral anticoagulation (any)***	3 (3.1)	163 (78.0)	< 0.001
*• Direct oral anticoagulant (DOAC)*	3 (3.1)	139 (66.5)	< 0.001
*• Vitamin K antagonist (VKA)*	0 (0.0)	24 (11.5)	< 0.001
***No antithrombotic therapy***	0 (0.0)	7 (3.3)	0.18

Values are presented in *n* (%), *ASA* = acetylsalicylic acid; *P2Y₁₂* = platelet adenosine diphosphate receptor inhibitor; *DOAC* = direct oral anticoagulant; *VKA* = vitamin K antagonist; *ESUS* = embolic stroke of undetermined source; *CES* = cardioembolic stroke

### Stroke severity 

Stroke severity at presentation was significantly lower among patients with ESUS than among those with CES. The mean NIHSS score on admission was 6 (95% CI, 5 to 7) in the ESUS group and 11 (95% CI, 10 to 12) in the cardioembolic group (*p* < 0.001). This difference persisted at discharge, with mean NIHSS scores of 2 (95% CI, 1 to 3) and 5 (95% CI, 4 to 6), respectively (*p* < 0.001). Among patients with ESUS, women tended to present higher stroke severity than men (mean NIHSS, 7 [95% CI, 6 to 8] vs. 5 [95% CI, 4 to 6]; *p* = 0.08), though the difference was not statistically significant. In contrast, among CES patients, women had significantly higher NIHSS scores at presentation than men (12 [95% CI, 11 to 13] vs. 10 [95% CI, 9 to 11]; *p* = 0.01). The presence of short-duration atrial fibrillation (< 30 s)^17^ in ESUS patients was associated with greater neurological impairment (mean NIHSS, 8 [95% CI, 7 to 9] vs. 6 [95% CI, 5 to 7]; *p* = 0.01) (Fig. 2b)

### Functional Outcome

**Fig. 3:**
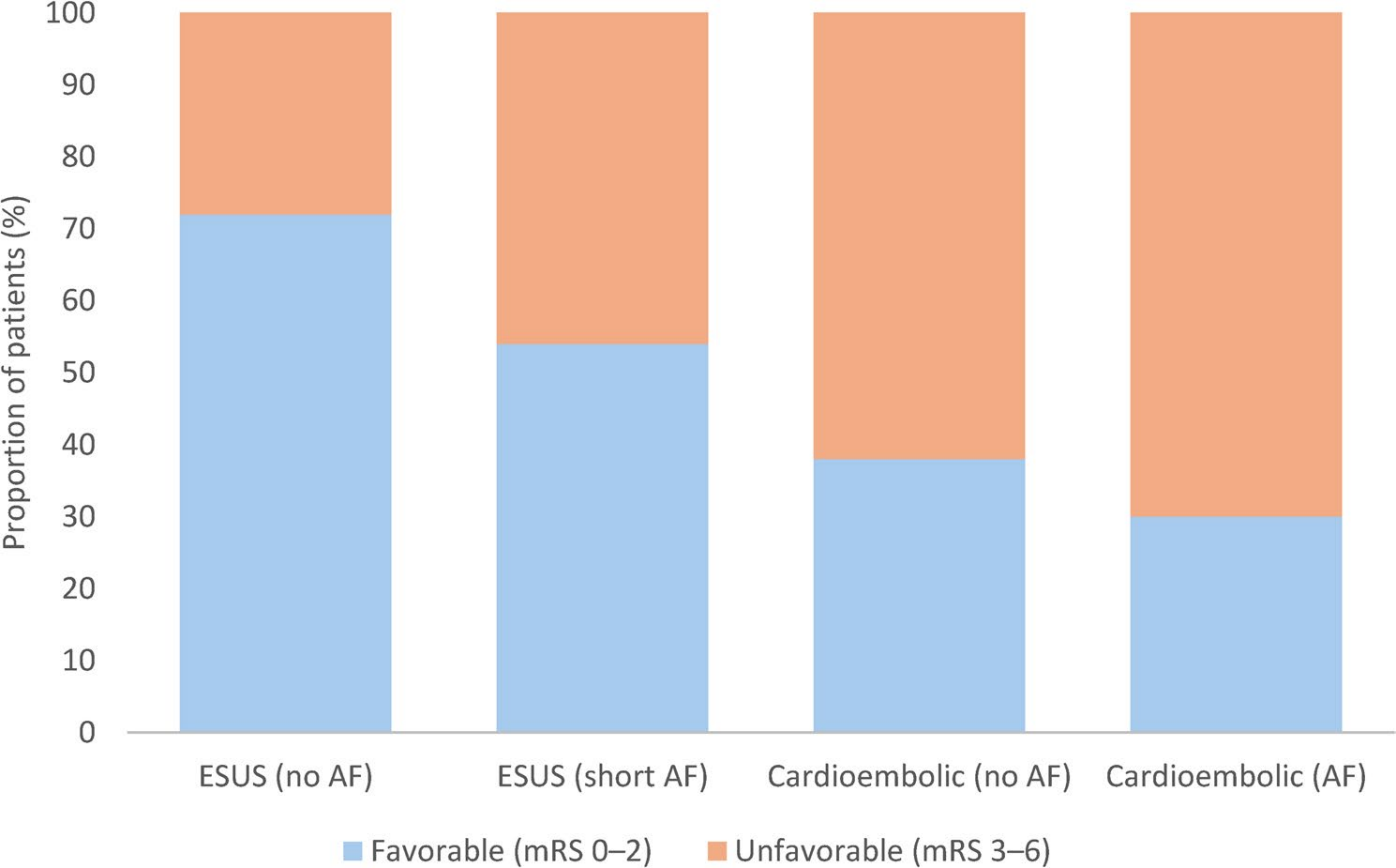
Distribution of functional outcomes by stroke subtype. Stacked bar chart showing the proportion of patients with favorable (0–2) versus unfavorable (3–6) modified Rankin Scale (mRS) outcomes at discharge across four groups: embolic stroke of undetermined source (ESUS) without atrial fibrillation (AF), ESUS with short-duration AF, cardioembolic stroke (CES) without AF, and CES with AF. Favorable outcomes decreased stepwise from ESUS without AF to CES with AF

**Fig. 4:**
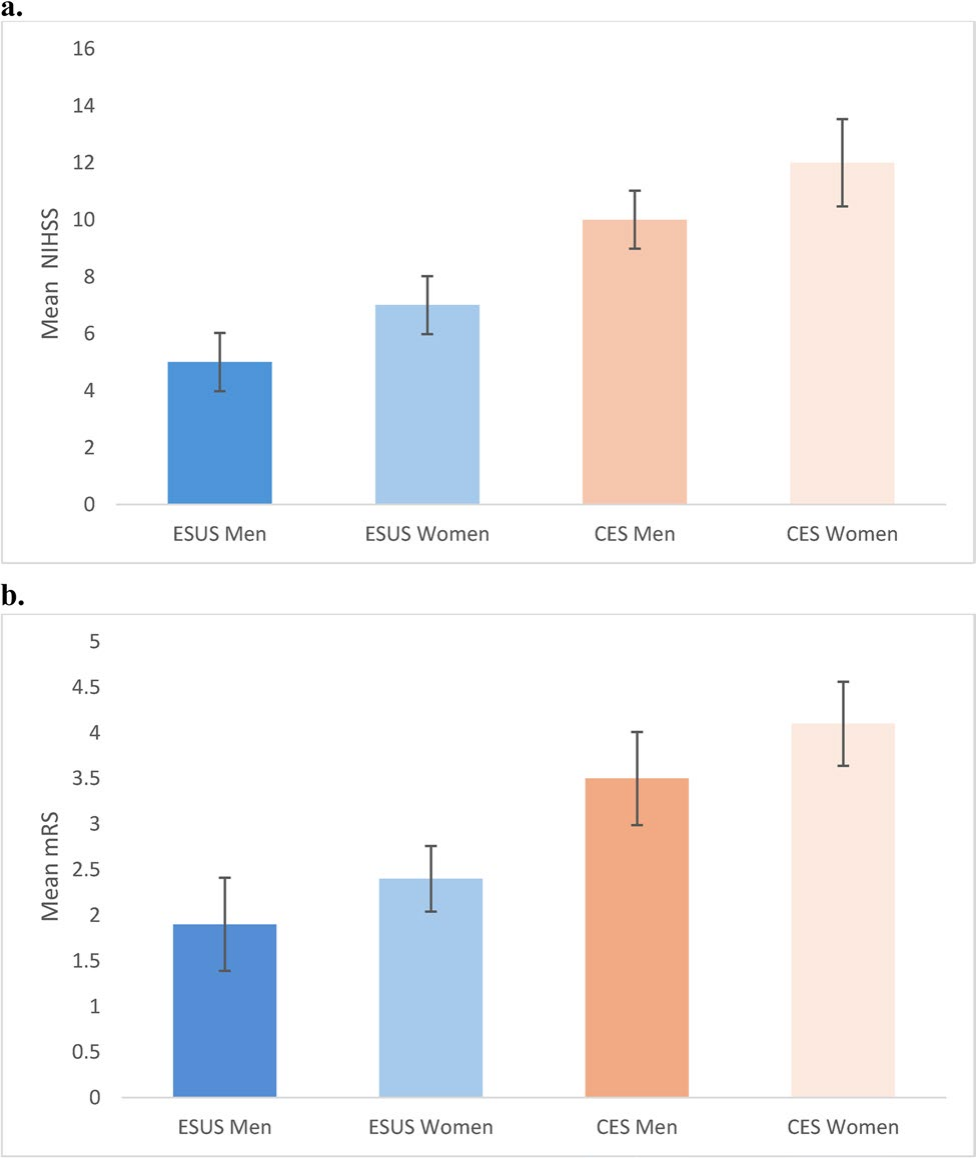
Stroke severity and functional outcome by sex and stroke subtype **A** Stroke Severity by Sex and Stroke Subtype. Bar plots show mean stroke severity at admission, measured by the National Institutes of Health Stroke Scale (NIHSS), stratified by sex and stroke subtype. Women had higher NIHSS scores than men across both stroke subtypes, with the highest severity observed in women with cardioembolic stroke (CES). **B** Functional Outcome at Discharge by Sex and Stroke Subtype. Bar plots depict mean modified Rankin Scale (mRS) scores at discharge, reflecting functional outcome, stratified by sex and stroke subtype. Women had worse functional outcomes than men in both stroke subtypes, with CES women showing the greatest disability

At discharge, functional outcomes measured by the mRS) were significantly better in the ESUS group than in the cardioembolic group. The mean mRS score was 2.1 (95% CI, 1.8 to 2.4) for ESUS and 3.8 (95% CI, 3.5 to 4.1) for CES (*p* < 0.001). A favorable functional outcome (mRS 0–2) was achieved in 68% of ESUS patients compared to 34% of cardioembolic patients (*p*< 0.001)(Fig. 3). In-hospital mortality was significantly lower in the ESUS group than in the cardioembolic group (3% vs. 11%; *p* = 0.004). Among patients with ESUS, women had higher mean mRS scores than men (2.4 [95% CI, 2.1–2.7] vs. 1.9 [95% CI, 1.6–2.2]; *p* = 0.09), although the difference was not statistically significant. In the cardioembolic group, female sex was associated with significantly poorer outcomes (mean mRS, 4.1 [95% CI, 3.8 to 4.4] vs. 3.5 [95% CI, 3.2 to 3.8]; *p* = 0.02) (Fig. 4). Among ESUS patients, short-duration atrial fibrillation was associated with worse functional outcomes (mean mRS, 2.8 [95% CI, 2.5 to 3.1] vs. 2.1 [95% CI, 1.8 to 2.4]; *p* = 0.02)

Nonetheless, when compared directly to CES patients, ESUS patients with short-duration atrial fibrillation still showed significantly lower stroke severity (mean NIHSS, 8 [95% CI, 7 to 9] vs. 11 [95% CI, 10 to 12]; *p * < 0.001) and better functional outcomes (mean mRS, 2.8 [95% CI, 2.5 to 3.1] vs. 3.8 [95% CI, 3.5 to 4.1]; *p* < 0.001), along with lower in-hospital mortality (3% vs. 11%; *p* = 0.01). In multivariable analysis adjusting for age, sex, vascular risk factors, and atrial fibrillation status, CES remained independently associated with greater stroke severity (adjusted β = +4.1 NIHSS points; *p* < 0.001) and worse functional outcome (adjusted β = +1.5 mRS points; *p *< 0.001).

**Fig. 5:**
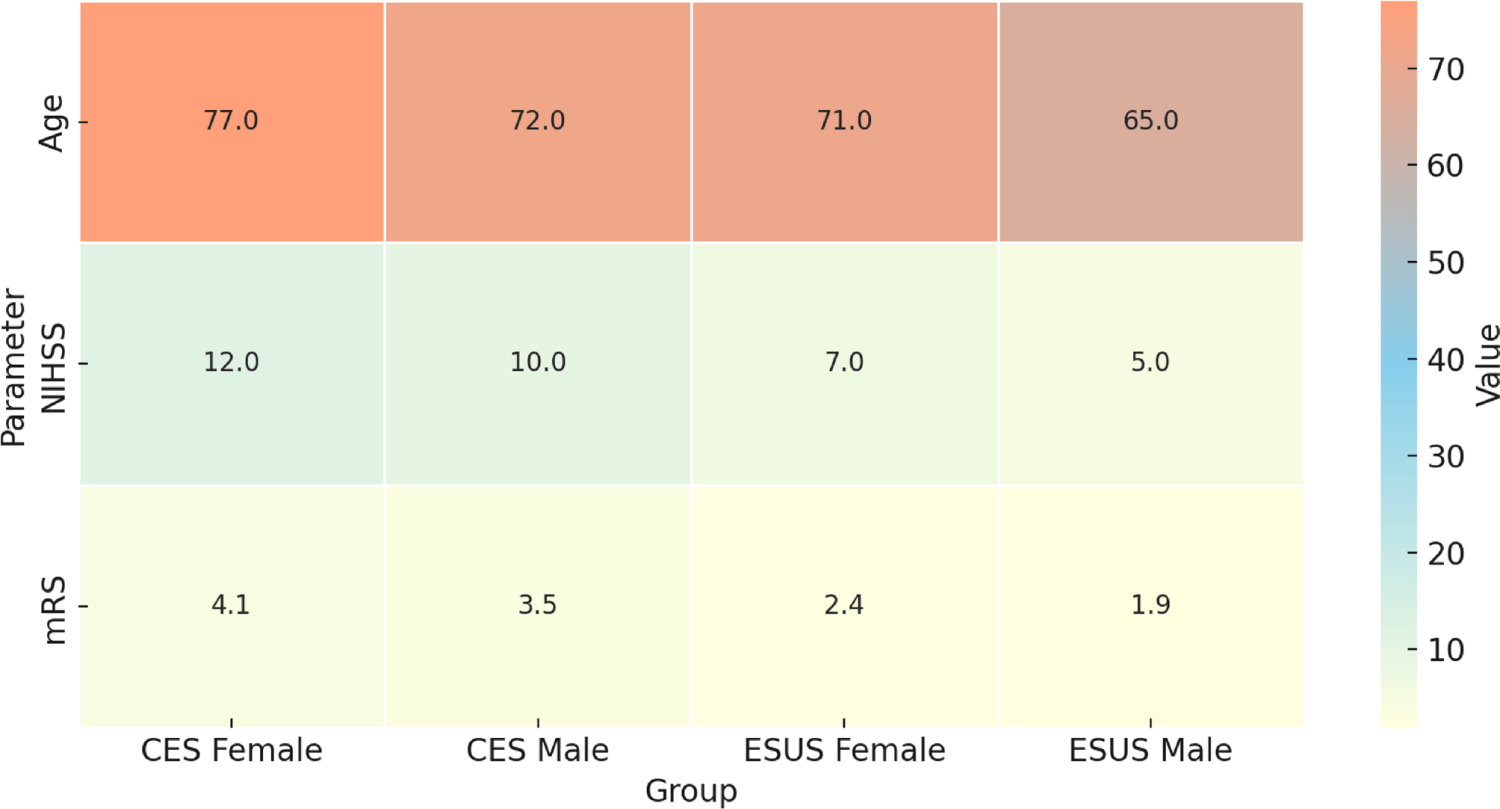
Age, stroke severity, and functional outcome by sex and stroke subtypeHeatmap displaying mean age, stroke severity (National Institutes of Health Stroke Scale [NIHSS]), and functional outcome (modified Rankin Scale [mRS]) in male and female patients with embolic stroke of undetermined source (ESUS) and cardioembolic stroke (CES). Female patients with CES were the oldest and had the highest NIHSS and mRS values, whereas male ESUS patients were the youngest and had the most favorable outcomes. The gradation of color intensity reflects the magnitude of each parameter, highlighting sex-based disparities in stroke presentation and recovery

No deaths occurred among ESUS patients during hospitalization. The in-hospital mortality rate among CES patients was 10.0% (21 of 209; *p* = 0.02). Among cardioembolic patients, women had a higher mortality rate than men (12.3% vs. 7.4%), although the difference did not reach statistical significance (*p* = 0.18). In multivariable analysis adjusting for age, sex, vascular risk factors, and atrial fibrillation status, CES remained independently associated with greater stroke severity (adjusted β = +4.1 NIHSS points; *p* < 0.001) and worse functional outcome (adjusted β = +1.5 mRS points; *p* < 0.001). The gradation of outcome severity by sex and stroke subtype is illustrated in Fig. 5.

### Age-related differences in stroke severity early outcome

Age distribution differed significantly between patients with ESUS and those with CES. Among ESUS patients, 36% were younger than 65 years, 49% were aged 65–79 years, and 15% were aged ≥ 80 years. In contrast, only 17% of CES patients were under 65 years, 45% were aged 65–79 years, and 37% were aged ≥ 80 years. This distribution was significantly different between groups (χ² = 16.6, *p* = 0.0003), confirming that ESUS disproportionately affects younger individuals, whereas CES is more prevalent in the elderly. Within the ESUS group, women were significantly older than men (mean age 71 ± 10.2 vs. 65 ± 9.6 years, *p* = 0.02), but sex-based differences in stroke severity and functional outcome did not reach statistical significance.

Stroke severity and early outcomes varied by both age and stroke subtype. In ESUS, patients aged < 65 years had the lowest mean NIHSS scores at discharge (1.3 ± 1.6), while those ≥ 80 years had significantly higher scores (3.2 ± 2.9; *p* = 0.01). A similar pattern was observed in CES, with younger patients (< 65 years) showing lower NIHSS scores (3.4 ± 2.9) compared to those ≥ 80 years (6.1 ± 4.1; *p* < 0.001). Functional independence (defined as NIHSS ≤ 5 at discharge) was achieved in 87% of younger ESUS patients (95% CI, 72.4–94.5%) compared to 61% of older CES patients (95% CI, 45.0–74.9%), with this difference reaching statistical significance *p* = 0.012).

### Adjusted analyses of stroke severity and early outcomes

In multivariable regression analyses adjusting for age, sex, and vascular risk factors, CES remained independently associated with greater neurological impairment and worse functional outcomes compared with ESUS. CES patients presented with significantly higher stroke severity at admission (adjusted β = +4.1 NIHSS points; 95% CI, 3.2–5.0; *p* < 0.001) and at discharge (adjusted β = +2.7 NIHSS points; 95% CI, 1.9–3.5; *p* < 0.001) (Table 2). Functional outcome at discharge was also worse in CES, with an adjusted difference of + 1.5 mRS points (95% CI, 1.1–1.9; *p* < 0.001). In logistic regression, CES was associated with more than a twofold higher risk of in-hospital mortality compared with ESUS (OR 2.4, 95% CI, 1.3–4.7; *p* = 0.004). Results were robust after adjustment for admission NIHSS, and sensitivity analysis restricted to NIHSS-matched subgroups yielded consistent findings (Table 5).

**Table 5:** Multivariable regression models comparing CES and ESUS. Linear regression models were used for NIHSS and mRS outcomes, logistic regression for in-hospital mortality. All models were adjusted for age, sex, hypertension, diabetes, hyperlipidemia, smoking status, and atrial fibrillation status. Admission NIHSS was additionally included in sensitivity analyses, which yielded consistent results

*Outcome*	*Predictor (CES vs. ESUS)*	*Adjusted β/OR (95% CI)*	*P-value*
*NIHSS at admission*	CES (ref = ESUS)	+4.1 (95% CI, 3.2–5.0)	<0.001
*NIHSS at discharge*	CES (ref = ESUS)	+2.7 (95% CI, 1.9–3.5)	<0.001
*mRS at discharge*	CES (ref = ESUS)	+1.5 (95% CI, 1.1–1.9)	<0.001
*In-hospital mortality (yes)†*	CES (ref = ESUS)	OR 2.4 (95% CI, 1.3–4.7)	0.004

*CES *Cardioembolic stroke*, ESUS *Embolic stroke of undetermined source*, mRS *modified Rankin Scale*, NIHSS *National Institutes of Health Stroke Scale

### 30-day outcomes

At 30 days after the index event, patients with ESUS continued to show more favorable outcomes compared with those with CES. Functional independence (mRS 0–2) was achieved in 62% of ESUS patients versus 29% of CES patients (*p* < 0.001). The mean mRS score at day 30 was 2.3 (95% CI, 2.0–2.6) in the ESUS group compared with 4.0 (95% CI, 3.7–4.3) in the CES group (*p* < 0.001). Thirty-day mortality remained significantly lower among ESUS patients (4.1%) than in CES patients (12.9%; *p*= 0.01) (Fig. 5) Sex-stratified analyses confirmed that CES women remained the subgroup with the poorest outcomes, with a mean mRS of 4.2 (95% CI, 3.8–4.6) and a mortality rate of 14.1%, compared with 10.7% in CES men (*p* = 0.18). In the ESUS group, outcomes between women and men did not differ significantly, although women showed a numerical trend toward higher disability (mean mRS, 2.6 vs. 2.1; *p* = 0.08). Age-related differences persisted across both subtypes. Younger ESUS patients (< 65 years) had the highest proportion of functional independence (74%), whereas older CES patients (≥ 80 years) had the lowest (22%). This interaction between stroke subtype and age was statistically significant (*p*= 0.01)

In multivariable regression analyses adjusting for age, sex, vascular risk factors, and atrial fibrillation status, CES remained independently associated with poorer 30-day outcomes (adjusted β for mRS = + 1.6, 95% CI 1.2–2.0; *p* < 0.001; adjusted OR for mortality = 2.7, 95% CI 1.4–5.2;*p*= 0.003)

### One-year outcomes

**Fig. 6:**
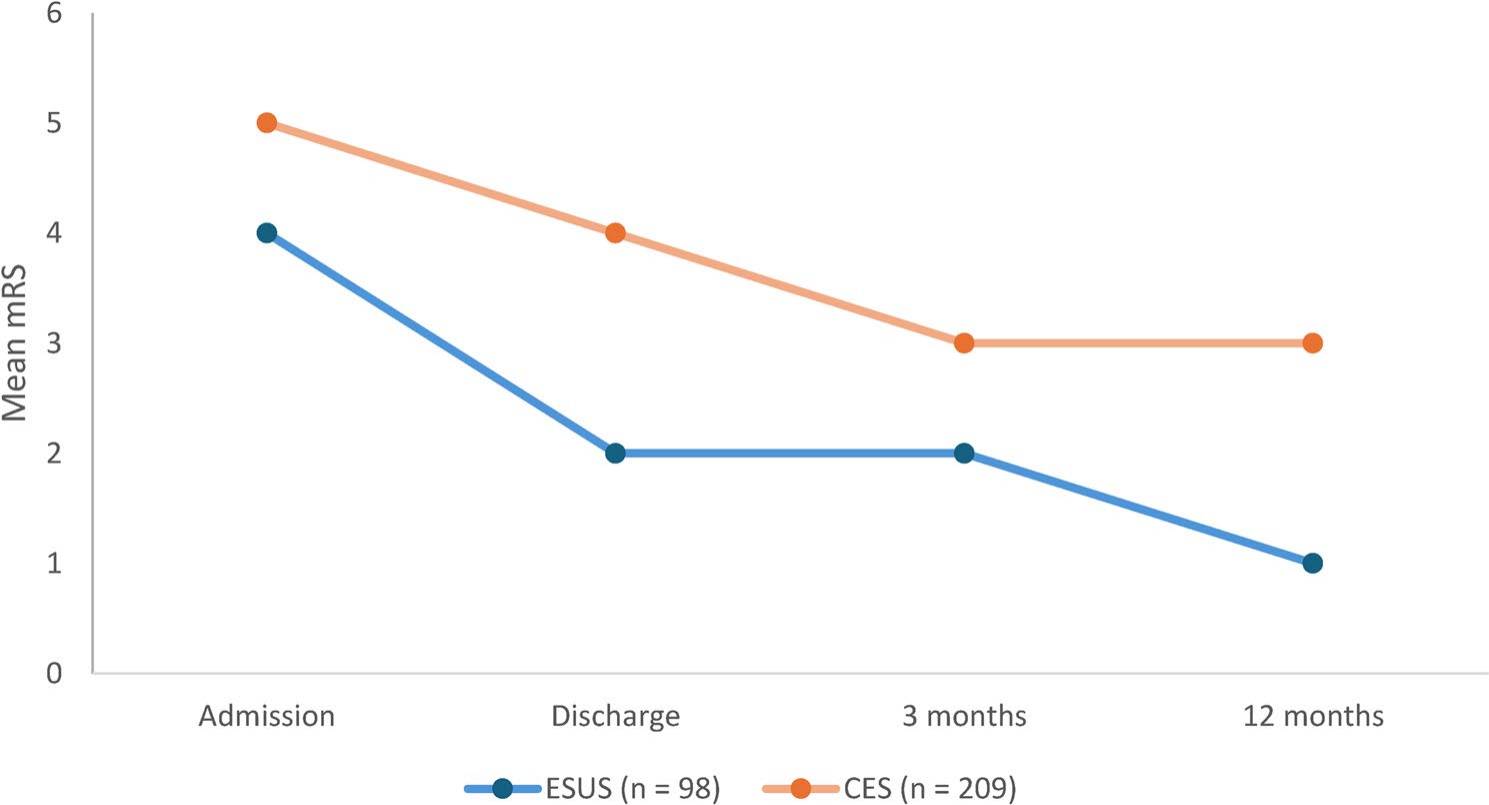
Longitudinal trajectory of functional outcomes by stroke subtype. Line chart showing mean modified Rankin Scale (mRS) scores in patients with embolic stroke of undetermined source (ESUS) and cardioembolic stroke (CES) from admission to 1-year follow-up. While both groups improved over time, ESUS patients consistently showed more favorable outcomes compared to CES patients

At 12 months, follow-up was completed in 245 of 307 patients (79.8%). Baseline age, sex, stroke subtype distribution, and admission NIHSS did not differ between completers and non-completers (Supplementary Table 2). ESUS patients continued to demonstrate more favorable long-term recovery compared with CES. Functional independence (mRS 0–2) was achieved in 60% of ESUS patients (95% CI, 51–69%) versus 28% of CES patients (95% CI, 22–34%; *p* < 0.001) (Fig. 6; Table 6). The mean NIHSS at 12 months was 1.7 (95% CI, 1.2–2.2) for ESUS and 4.6 (95% CI, 3.9–5.3) for CES (*p* < 0.001).

**Table 6:** Longitudinal functional outcomes in patients with ESUS and CES. Comparison of NIHSS, mRS, and mortality at admission, discharge, 3 months, and 12 months. ESUS patients showed milder neurological deficits and better recovery trajectories across all time points

***Outcome***	***Time point***	***ESUS (n = 98)***	**CES (n = 209)**	***P value***
***NIHSS, median (IQR)***	Admission	6 (3–11)	12 (7–18)	**< 0.001**
	Discharge	2 (1–5)	6 (2–13)	**< 0.001**
	3 months	2 (1–4)	5 (2–10)	**0.001**
	12 months	1 (0–3)	4 (1–7)	**0.002**
***mRS, median (IQR)***	Admission	4 (3–5)	5 (4–5)	**0.001**
	Discharge	2 (1–3)	4 (2–4)	**< 0.001**
	3 months	2 (1–3)	3 (2–4)	**0.002**
	12 months	1 (0–2)	3 (1–4)	**< 0.001**
***Mortality, n (%)***	30 days	4 (4.1 %)	26 (12.4 %)	**0.019**
	3 months	6 (6.1 %)	33 (15.8 %)	**0.008**
	12 months	7 (7.1 %)	41 (19.6 %)	**0.004**

Continuous variables (NIHSS, mRS) are presented as median (IQR) and compared using Mann–Whitney U tests; categorical variables (mortality) are presented as n (%) and compared using χ² tests

*CES* Cardioembolic stroke, *ESUS* Embolic stroke of undetermined source, *mRS* modified Rankin Scale, *NIHSS* National Institutes of Health Stroke Scale

While both groups showed progressive improvement within the first three months, functional recovery in CES reached a plateau thereafter, indicating that most neurological gains occurred during the subacute phase. In contrast, ESUS patients exhibited continued improvement throughout the first year, consistent with their lower initial stroke severity and preserved neural reserve. Cumulative one-year mortality remained significantly lower in ESUS (7.1%) than in CES (21.5%; *p* < 0.001). Women with CES showed the poorest long-term outcomes, with only 24% achieving functional independence at 12 months, compared with 33% in men ( *p* = 0.05). In ESUS, sex-based differences were less pronounced (57% vs. 63%; *p*= 0.32). Age remained a strong modifier, with younger ESUS patients (< 65 years) achieving the most favorable recovery (78% with mRS 0–2 at 12 months). At 12 months, mean EQ-5D index scores were significantly higher in ESUS compared with CES (0.78 ± 0.15 vs. 0.61 ± 0.18; *p*< 0.001), and Barthel Index scores similarly favored ESUS (87 ± 14 vs. 69 ± 21;*p* < 0.001). The domains most affected in CES were mobility and self-care. No significant sex differences were observed within ESUS, whereas women with CES reported lower EQ-5D scores than men (0.57 vs. 0.64; *p* = 0.04). A longitudinal comparison of functional outcomes between ESUS and CES is presented in Table 6. Across all time points, ESUS patients demonstrated significantly lower NIHSS and mRS scores and lower mortality rates, indicating a milder initial presentation and steeper recovery trajectory compared with CES.

### Sensitivity analyses including baseline NIHSS

In sensitivity analyses including baseline NIHSS as a covariate in the multivariable logistic regression model, the association between stroke subtype and functional outcome remained directionally consistent and statistically significant (adjusted OR 1.72, 95% CI 1.05–2.83; *p*= 0.031). Subgroup analyses stratified by baseline stroke severity (NIHSS ≤ 5 vs. > 5) yielded similar trends, indicating that initial stroke severity only partially accounted for the observed differences between ESUS and CES.

## Discussion

This study demonstrates that ESUS is not merely a milder variant of cardioembolism, but a distinct clinical entity with its own recovery dynamics. By capturing this gradient of severity and outcome, our data bridge the ongoing debate about whether ESUS represents a truly cryptogenic form or part of an evolving cardioembolic spectrum. In this prospective cohort study, patients ESUS presented with significantly milder neurological deficits and achieved more favorable functional outcomes at hospital discharge than those with CES [31]. These early differences persisted even among ESUS patients with short-duration AF, who exhibited an intermediate phenotype—worse outcomes than ESUS without AF, but better than CES [32]. Together, these findings highlight the distinct, and heterogeneous, clinical course of ESUS and support the need for a more nuanced approach to its classification and management [33].

The relatively benign presentation of ESUS aligns with prior observational data. In a Mexican cohort, Arauz et al. reported that ESUS patients were not only significantly younger but also experienced lower mortality and better long-term outcomes compared to those with CES [34]. Similarly, a large population-based study demonstrated that ESUS patients had significantly lower NIHSS scores at admission and better outcomes at multiple timepoints up to one year post-stroke [35]. In the Athens Stroke Registry, Ntaios et al. observed that although stroke recurrence rates were similar between ESUS and cardioembolic groups, ESUS was associated with a markedly higher cumulative survival probability (65.6% vs. 38.8%) [36]. Our data confirm and extend these findings in a prospective hospital-based setting. At discharge, nearly 70% of ESUS patients achieved functional independence (mRS 0–2), compared with only one-third of patients with cardioembolic stroke. These associations remained directionally consistent after adjustment for baseline NIHSS, indicating that initial stroke severity only partially accounts for the outcome differences between ESUS and CES. In-hospital mortality was more than threefold lower in ESUS (3% vs. 11%). These early outcome differences likely reflect both clinical severity and pathophysiological mechanisms. The embolic burden in ESUS may be lower, and its sources more transient or less thrombogenic than in CES, where sustained atrial arrhythmia and thrombus formation confer high embolic potential [33] Our findings highlight important demographic modifiers of stroke outcomes across embolic subtypes. Age emerged as a major determinant of stroke severity and early recovery. ESUS patients were significantly younger than those with CES, and younger age was consistently associated with lower NIHSS scores and higher rates of early functional independence. This age gradient may partially account for the milder clinical profile observed in ESUS. Sex-specific differences also emerged in our cohort. Women with CES were significantly older and experienced more severe strokes and poorer functional outcomes compared to men, consistent with prior reports of increased thrombus burden and age-related comorbidity in female CES patients [37, 38]. These results parallel recent analyses indicating that female sex independently modifies stroke severity and recovery, even after adjustment for age, comorbidity, and anticoagulation patterns, pointing toward intrinsic biological and hemodynamic contributors to poorer outcomes in women [39]. Among ESUS patients, women were also older and showed a trend toward higher NIHSS scores and worse recovery than men, although these differences did not reach statistical significance—likely reflecting limited subgroup size and the milder overall presentation in ESUS. Collectively, these findings suggest that both age and sex interact with stroke subtype to influence early clinical outcomes and should be considered in future ESUS classification and risk stratification efforts [39].

Our exploratory analysis provides further insight into the underlying embolic substrates of ESUS and CES. Despite exclusion of major cardioembolic sources, nearly half of ESUS patients exhibited minor or latent abnormalities such as non-occlusive atherosclerotic plaques [40–42], atrial cardiopathy [43, 44], or brief atrial arrhythmias [17, 45]. These findings indicate that ESUS encompasses a broad pathophysiological spectrum rather than a single entity. The overlap of such subclinical sources with those observed in CES supports the concept of a mechanistic continuum—from covert embolic susceptibility to overt cardioembolism—which may contribute to the observed gradient in stroke severity and functional outcomes. This perspective aligns with emerging evidence emphasizing refined etiologic stratification and extended rhythm monitoring to uncover concealed embolic mechanisms [46]. In line with this continuum, recent hemodynamic studies by Chen et al. [47, 48] demonstrated that alterations in wall shear stress and translesional pressure gradients are closely linked to plaque vulnerability and infarct pattern and typology [49, 50], supporting the interplay between local vascular mechanics and embolic stroke risk. Complementing these vascular mechanisms, circulating markers such as D-dimer, BNP/NT-proBNP, CK-MB and troponin have been associated with ESUS and covert cardioembolic sources, and may refine etiologic probability when imaging findings are inconclusive [51, 52].These vascular mechanisms parallel our observations on atrial dynamics, highlighting converging arterial and atrial pathways of embolic susceptibility in ESUS. Another key finding of our study is the intermediate risk profile of ESUS patients with short-duration atrial fibrillation (episodes < 30 s). By current guideline criteria, such brief arrhythmias do not meet the diagnostic threshold for atrial fibrillation and therefore do not reclassify patients as CES. Nonetheless, these episodes may reflect underlying atrial cardiopathy and a predisposition to future embolic events rather than a benign finding [17]. In our cohort, ESUS patients with short-duration AF had more severe strokes than those without AF, yet outcomes remained superior to those with overt cardioembolic sources. This pattern supports the concept of atrial cardiopathy as a continuum, where even brief arrhythmias may indicate a substrate prone to thromboembolism [53]. Prior research has shown that subclinical AF often emerges months or years after an index ESUS event [45], raising the possibility that many ESUS cases are misclassified due to insufficient monitoring [54, 55]. The pronounced divergence in antithrombotic regimens at discharge further reinforces this mechanistic continuum [56]. While nearly all ESUS patients were managed with single antiplatelet therapy, oral anticoagulation remained the mainstay in CES, reflecting the transition from latent to manifest cardioembolism. These therapeutic differences mirror underlying pathophysiology rather than treatment bias [57]. Thus, the more favorable outcomes observed in ESUS are unlikely to be therapy-mediated but instead reflect a lower embolic burden and the transient or subclinical nature of its embolic substrates. This mechanistic gradient has direct therapeutic implications. It suggests that current categorical definitions may insufficiently capture evolving embolic risk, particularly in ESUS patients with recurrent brief arrhythmias or atrial remodeling. Randomized trials such as NAVIGATE ESUS [2] and RE-SPECT ESUS [3] failed to demonstrate a benefit of empiric anticoagulation in unselected ESUS populations. However, our results suggest that certain high-risk phenotypes—such as ESUS with recurrent, brief arrhythmias—might benefit from rhythm-guided or biomarker-enhanced secondary prevention strategies [8, 47, 58, 59].

This aligns with emerging proposals to tailor treatment not solely by stroke subtype, but by evolving risk profile, including cardiac and aortic abnormalities, imaging, and electrophysiological markers [48, 60]. Recent multicenter data further support this concept, showing that subgroups such as ESUS with left ventricular injury may derive greater benefit from anticoagulation compared with antiplatelet therapy [58].

Beyond the acute phase, our follow-up data demonstrate that outcome differences between ESUS and CES persist at 30 days and remain evident at 1 year. ESUS patients not only had lower disability scores and mortality during hospitalization, but also maintained higher rates of functional independence and survival in the subacute and long-term phase. These findings are in line with population-based data reporting superior cumulative survival in ESUS compared with CES [36, 61]. The persistence of this prognostic gap highlights that ESUS is not merely an attenuated form of CES, but rather a distinct clinical entity with a more favorable natural history [34]. Importantly, the observed sex- and age-related gradients also extended into the 30-day and 1-year follow-up, with older CES women remaining the most vulnerable subgroup, whereas younger ESUS patients preserved the highest functional recovery rates [62].

In summary, our findings underscore the heterogeneity of ESUS, both clinically and demographically. While some ESUS cases may represent covert CES, the overall milder presentation and demographic profile—despite infarct patterns often resembling CES—suggest that ESUS comprises a distinct and heterogeneous spectrum requiring tailored diagnostic and therapeutic approaches. Recognizing this spectrum reframes ESUS as a dynamic vascular disorder rather than a residual diagnostic category. Future studies should therefore focus on defining mechanistic subgroups and individualized prevention strategies that reflect this evolving concept.

### Limitations

This study has several limitations. It was conducted in a single-center, observational design, which may restrict external validity. The stroke unit operates within Germany’s universal healthcare system and serves as the primary referral center for both urban and rural populations across an extensive catchment area, with the next comprehensive stroke facility located more than 80 km away. This organizational structure likely yielded a demographically and clinically representative regional cohort; however, variations in population composition and healthcare systems across countries may limit the broader generalizability of our findings. We acknowledge that baseline stroke severity (NIHSS) influences functional outcome; however, it was not included in the primary model to avoid overadjustment, as it represents an intermediate variable on the causal pathway between stroke subtype and outcome.

Moreover, the sample size, while prospectively recruited, limits statistical power for certain subgroup analyses. Extended outpatient cardiac monitoring beyond 48 h was not uniformly performed, which may have led to underestimation of the true prevalence of paroxysmal AF. Follow-up was completed using a combination of in-person visits, mailed questionnaires, and structured telephone interviews; this mixed approach may introduce reporting bias. Finally, the analyses are exploratory and hypothesis-generating, and external validation in larger multicenter cohorts will be required. Nevertheless, the study benefits from its prospective design, standardized diagnostic workup, and detailed phenotyping of embolic stroke subtypes, enabling a nuanced exploration of early prognostic patterns. Infarction pattern and biomarker data were not yet analyzed in the present study but are planned for evaluation in ongoing neuroradiological and translational substudies.

## Conclusion

In this prospective cohort, patients with ESUS presented with milder neurological deficits and had more favorable functional recovery than those with CES. These differences may be related to the heterogeneous and incompletely characterized mechanisms underlying ESUS, which are likely associated with a lower thromboembolic burden. Recognition of the prognostic divergence between ESUS and CES could contribute to refinement of stroke subtype classification. However, these findings should be considered exploratory and require confirmation in larger, multicenter cohorts to determine their implications for individualized secondary prevention strategies.

## Supplementary Information

Supplementary Material 1.

Supplementary Material 2.

## Data Availability

The datasets generated and/or analyzed during the current study are not publicly available due to institutional data protection regulations but are available from the corresponding author on reasonable request and after approval by the institutional data-protection officer.

## Abbreviations

AF: Atrial fibrillation
AAA: Aortic arch atherosclerosis
ACC: American College of Cardiology
AHA/ASA: American Heart Association/ American Stroke Association
ASA: Acetylsalicylic acid (Aspirin)
APHRS: Asia Pacific Heart Rhythm Society
BMI: Body mass index
BNP / NT-proBNP: B-type natriuretic peptide / N-terminal pro-B-type natriuretic peptide
CAD: Coronary artery disease
CES: Cardioembolic stroke
CI: Confidence interval
CK-MB: Creatine kinase - MB
CT: Computed tomography
CTA: Computed tomography angiography
DAPT: Dual antiplatelet therapy
DOAC: Direct oral anticoagulant
DWI: Diffusion-weighted imaging
ECAS: European Cardiac Arrhythmia Society
ECG: Electrocardiogram
EHRA: European Heart Rhythm Association
EQ-5D-5L: EuroQol 5 Dimensions 5 Levels
ESUS: Embolic stroke of undetermined source
HRS: Heart Rhythm Society
IQR: Interquartile range
LA: Left atrium
LAHRS: Latin American Heart Rhythm Society
LAVI: Left atrial volume index
LVEF: Left ventricular ejection fraction
MRI: Magnetic resonance imaging
MRA: Magnetic resonance angiography
mRS: Modified Rankin Scale
NASCET: North American Symptomatic Carotid Endarterectomy Trial (Criteria)
NIHSS: National Institutes of Health Stroke Scale
NOAC: Non-vitamin K oral anticoagulant (falls bewusst verwendet; sonst konsistent nur DOAC nutzen)
OR: Odds ratio
PFO: Patent foramen ovale
P2Y12: Purinergic receptor P2Y12 inhibitor (z. B. Clopidogrel/Ticagrelor/Prasugrel)
SUM: Stroke unit monitoring
SD: Standard deviation
TEE: Transesophageal echocardiography
TIA: Transient ischemic attack
TOAST: Trial of Org 10,172 in Acute Stroke Treatment (Classification)
TTE: Transthoracic echocardiography
VKA: Vitamin K antagonist
