# Supplementary material for: Cardioembolic stroke versus embolic stroke of undetermined source: early severity and long-term outcomes in a prospective cohort
Source: BMC Neurol. 2025 Dec 1;25:503. doi: 10.1186/s12883-025-04494-w (PMC12701600; doi:10.1186/s12883-025-04494-w)
Supplement: Supplementary file 1 — Supplementary Material 1. [file 12883_2025_4494_MOESM1_ESM.docx]

**Supplementary Table 1. Neurological severity and functional outcome in ESUS and CES patients across follow-up**

| **Time point** | **NIHSS median (IQR)** | **p-value** | **mRS median (IQR)** | **p-value** | **Mortality n (%)** | **p-value** |
| --- | --- | --- | --- | --- | --- | --- |
| **Admission** | ESUS 3 (2–6) | < 0.001 | 4 (3–5) | < 0.001 | 5 (5.1) | 0.04 |
|  | CES 8 (4–14) |  | 5 (3–6) |  | 31 (14.8) |  |
| **Discharge** | ESUS 1 (0–3) | < 0.001 | 2 (1–3) | < 0.001 | 6 (6.1) | 0.03 |
|  | CES 4 (2–8) |  | 3 (2–5) |  | 44 (21.1) |  |
| **30 days** | ESUS 1 (0–2) | 0.001 | 1 (0–2) | 0.002 | 7 (7.1) | 0.05 |
|  | CES 3 (1–5) |  | 2 (1–4) |  | 49 (23.4) |  |
| **12 months** | ESUS 0 (0–1) | 0.004 | 1 (0–2) | 0.006 | 10 (10.2) | 0.04 |
|  | CES 2 (1–4) |  | 2 (1–4) |  | 55 (26.3) |  |

Values are expressed as median (interquartile range) or n (%). NIHSS = National Institutes of Health Stroke Scale; mRS = modified Rankin Scale. p-values derived from Mann–Whitney U test for continuous variables and χ² test for categorical variables.
