# Supplementary material for: Cardioembolic stroke versus embolic stroke of undetermined source: early severity and long-term outcomes in a prospective cohort
Source: BMC Neurol. 2025 Dec 1;25:503. doi: 10.1186/s12883-025-04494-w (PMC12701600; doi:10.1186/s12883-025-04494-w)
Supplement: Supplementary file 2 — Supplementary Material 2. [file 12883_2025_4494_MOESM2_ESM.docx]

**Supplementary Table 2. Baseline characteristics of patients with and without completed 12-month follow-up**

| **Variable** | **Follow-up completed (n = 245)** | **Lost to follow-up (n = 62)** | **p-value** |
| --- | --- | --- | --- |
| Age, years (mean ± SD) | 71.8 ± 10.9 | 72.6 ± 11.4 | 0.59 |
| Female sex, n (%) | 118 (48.2) | 29 (46.8) | 0.85 |
| Stroke subtype, n (%) |  |  | 0.67 |
| – ESUS | 78 (31.8) | 20 (32.3) |  |
| – CES | 167 (68.2) | 42 (67.7) |  |
| NIHSS at admission, median [IQR] | 6 [3–11] | 7 [3–12] | 0.48 |
| Hypertension, n (%) | 189 (77.1) | 47 (75.8) | 0.83 |
| Diabetes mellitus, n (%) | 72 (29.4) | 20 (32.3) | 0.68 |
| Hyperlipidemia, n (%) | 131 (53.5) | 31 (50.0) | 0.65 |
| Atrial fibrillation, n (%) | 88 (35.9) | 22 (35.5) | 0.95 |
| BMI, kg/m² (mean ± SD) | 26.8 ± 3.9 | 26.6 ± 4.1 | 0.74 |

Categorical variables were compared using χ² or Fisher’s exact tests, and continuous variables with t-tests or Mann–Whitney U tests, as appropriate. No significant baseline differences were observed between patients with and without 12-month follow-up.
